# Coexistence of two quadruplex–duplex hybrids in the *PIM1* gene

**DOI:** 10.1093/nar/gkaa752

**Published:** 2020-09-25

**Authors:** Derrick J Y Tan, Fernaldo Richtia Winnerdy, Kah Wai Lim, Anh Tuân Phan

**Affiliations:** School of Physical and Mathematical Sciences, Nanyang Technological University, Singapore 637371, Singapore; School of Physical and Mathematical Sciences, Nanyang Technological University, Singapore 637371, Singapore; School of Physical and Mathematical Sciences, Nanyang Technological University, Singapore 637371, Singapore; School of Physical and Mathematical Sciences, Nanyang Technological University, Singapore 637371, Singapore; NTU Institute of Structural Biology, Nanyang Technological University, Singapore 636921, Singapore

## Abstract

The triple-negative breast cancer (TNBC), a subtype of breast cancer which lacks of targeted therapies, exhibits a poor prognosis. It was shown recently that the *PIM1* oncogene is highly related to the proliferation of TNBC cells. A quadruplex–duplex hybrid (QDH) forming sequence was recently found to exist near the transcription start site of *PIM1*. This structure could be an attractive target for regulation of the *PIM1* gene expression and thus the treatment of TNBC. Here, we present the solution structures of two QDHs that could coexist in the human *PIM1* gene. Form 1 is a three-G-tetrad-layered (3+1) G-quadruplex containing a propeller loop, a lateral loop and a stem-loop made up of three G•C Watson–Crick base pairs. On the other hand, Form 2 is an anti-parallel G-quadruplex comprising two G-tetrads and a G•C•G•C tetrad; the structure has three lateral loops with the middle stem-loop made up of two Watson-Crick G•C base pairs. These structures provide valuable information for the design of G-quadruplex-specific ligands for *PIM1* transcription regulation.

## INTRODUCTION

The triple-negative breast cancer (TNBC), a subtype of breast cancer which lacks of targeted therapies, exhibits a poor prognosis (1). Recently it has been shown that the *PIM1* oncogene is overexpressed in TNBC as compared to receptor-positive breast cancers, and that PIM1 inhibition is lethal to MYC-overexpressing subpopulations (2,3). *PIM1* belongs to the proviral insertion site of Moloney murine leukemia virus (PIM) family of serine/threonine kinases that promote cellular survival and proliferation upon growth factor and cytokine signaling (4,5). *PIM1*, the first member to be discovered, was identified as an oncogene when its gene locus was found to be a frequent integration site in murine leukemia virus-induced lymphomas (6). PIM1 kinase is constitutively active, and its tumorigenic property in concert with MYC has been highlighted in hematopoietic and prostate cancers (4,7,8). As *PIM1*-knockout mice were shown to be viable with smaller body stature (9,10), inhibition of PIM1 kinase would thus represent an attractive anticancer treatment (3–5,11). Small-molecule inhibitors of PIM1 kinase are still in early clinical development phase (12) with limited success to date due to toxicity and tumor resistance (13), hence alternative approaches toward its inhibition/downregulation will be highly desirable.

In contrast to direct small-molecule binding of oncogenic proteins for inhibition, targeting of G-quadruplex (G4) structures represents an alternative approach of selective gene modulation at the transcriptional level (14). G4s are four-stranded nucleic acid structures formed by the assembly of multiple G•G•G•G tetrads (15–18). G4-forming sequences are prevalent in the human genome (19,20), with over 700 000 potential sites having been experimentally mapped (21). G4s were detected in ciliate (22) and human cells (23–25), and they have been implicated in various biological processes (26,27). In particular, G4-forming sequences were found to be enriched in oncogenic promoters, such as *MYC* (28), *KIT* (29) and *BCL2* (30), wherein small-molecule interventions were shown to downregulate transcription (31–33). Previously, we have shown that G4 and duplex structural elements can readily combine to give rise to stable quadruplex–duplex hybrids (QDHs) (34–36), and that such sequence motifs can be found across regulatory important regions in the human genome, including the *PIM1* gene (37). It was shown that duplex formation could guide, drive and accelerate adjacent G4 folding (34–36,38–43).

Here, we present the structures of two coexisting QDHs that can be formed in the natural *PIM1* sequence context. Form 1 comprises a three-G-tetrad (3+1) core and a duplex stem nested within the wide groove in a continuous stacking arrangement; Form 2 comprises a two-G-tetrad chair-type core and a duplex stem that extends outwards from the wide groove, with the G•C base pair immediately adjacent of the G-tetrad core further taking part in the establishment of an additional G•C•G•C tetrad. The two QDH structures provide structural elements for both sequence-specific (duplex-binding) and scaffold-specific (quadruplex-binding) targeting of such motifs for the selective modulation of gene expression.

## MATERIALS AND METHODS

### Sample preparation

Non-labeled DNA oligonucleotides were purchased from IDT Singapore. Site-specific low-enrichment (2%) ^15^N-labeled DNA oligonucleotides were chemically synthesized using an ABI 394 DNA/RNA synthesizer. All samples were dialyzed successively against ∼20 mM KCl solution and against deionized water before they were lyophilized. Unless otherwise specified, the oligonucleotides were dissolved in a buffer containing 20 mM KCl and 20 mM potassium phosphate, pH 7.

### NMR spectroscopy

NMR experiments were performed on a 600-MHz Bruker spectrometer. The strand concentration of the NMR samples was typically 0.1–2 mM. Data were recorded at 25°C, unless otherwise specified. NOESY, TOCSY, COSY and ^13^C–^1^H-HSQC spectra were recorded. Spectral analysis was performed using the SPARKY program (44). For 2D NOESY experiments, 2048 points were accumulated in the direct (F2) dimension with 600 (in 90%/10% H_2_O/D_2_O solvent) or 850 (in 100% D_2_O solvent) increments in the indirect (F1) dimension. The spectral widths are ∼20 ppm (in 90%/10% H_2_O/D_2_O solvent) or ∼10 ppm (in 100% D_2_O solvent) for both dimensions. The processing parameters are 4096 points for F2 and 2048 points for F1, with SINE window function and Sine bell shift (SSB) of 2 in both dimensions, unless otherwise stated.

### CD spectroscopy

The circular dichroism (CD) experiments were performed on a Jasco J-815 spectropolarimeter. The DNA strand concentration of the samples was typically 3-5 μM. The buffer contained 20 mM KCl and 20 mM potassium phosphate, pH 7. Samples were heated up and subsequently cooled in ice before CD measurement. The spectrum of the buffer was subtracted and the average of three scans was taken. For CD melting experiments, cooling and heating were successively performed across the temperature range of 15–95°C at a ramp rate of 0.5°C/min. The full spectrum was recorded at intervals of 1°C, after which the molar ellipticity at 295 nm was extracted. Two baselines corresponding to the completely folded (low temperature) and completely unfolded (high temperature) states were manually drawn. The melting temperature (*T_m_*) is defined as the temperature at which there are equal fractions of folded and unfolded species. The difference between the *T_m_* values from the folding and unfolding experiments for all quadruplex–duplex hybrids was less than 1.0°C.

### Gel electrophoresis

Gel electrophoresis was performed on a 10 cm × 7 cm native gel containing 20% acrylamide (acrylamide:bis-acrylamide = 37.5:1) with a running buffer containing 10 mM KCl in TBE (pH 8.3) at 120 V for 100 min. The gel was visualized by UV shadowing.

### Structure calculation

#### NOE distance restraints

Inter-proton distances for *PIM1 SLQS07* (Form 1) and *PIM1 SLQS02* (Form 2) were obtained from NOESY experiments performed in 90%/10% H_2_O/D_2_O and 100% D_2_O at various mixing times (100, 200 and 300 ms). For non-exchangeable protons, the peaks were classified as strong, medium, and weak, corresponding to the distance restraints of 2.7 ± 0.8, 3.8 ± 0.9 and 5.5 ± 1.7 Å, respectively. Distances from exchangeable protons were classified as strong, medium and weak, corresponding to the distance restraints of 4.0 ± 1.0, 4.8 ± 1.4 and (5.5 ± 1.7) Å, respectively. Distances involving thymine methyl protons were altered to be directed towards the respective methyl carbons with 0.5 Å looser restraints as compensation.

#### Dihedral restraints

Dihedral angle restraints were imposed to the dihedral angle formed by O4′–C1′–N9–C4 of guanine residues and O4′–C1′–N1–C2 of cytosine residues. *Anti* residues were restricted to an angle of (240 ± 70)° or (240 ± 40)° depending on the position of the base, while *syn* residues were restricted to an angle of (60 ± 70)° or (60 ± 40)°.

#### Hydrogen-bond restraints

Hoogsteen hydrogen bonds between guanines were restrained using H21–N7, N2–N7, H1–O6 and N1–O6 distances, which were set to 2.0 ± 0.2, 2.9 ± 0.3, 2.0 ± 0.2 and 2.9 ± 0.3 Å, respectively. The hydrogen bond restraints from the Watson-Crick interaction between guanine and cytosine were set to 2.0 ± 0.2 Å for H21–O2, H1–N3 and O6–H41, and 2.9 ± 0.3 Å for N2–O2, N1–N3 and O6–N4.

#### Planarity restraints

Planarity restraints were used for all the G-tetrads, G•C•G•C tetrads and G•C base pair in both structures.

#### Distance-geometry simulated-annealing

Initial extended conformations of both sequences were generated using the XPLOR-NIH program (45) by supplying the available standard DNA topology and parameter tables. Each system was then subjected to distance geometry simulated annealing by incorporating distance, dihedral, hydrogen-bond and planarity restraints. One hundred structures were generated and subjected to further refinement.

#### Distance-restrained molecular dynamics refinement

The 100 structures obtained from each simulated annealing step were refined with a distance-restrained molecular dynamics protocol incorporating all distance restraints. For each structure, the system was heated from 300 to 1000 K in 14 ps and allowed to equilibrate for 6 ps, during which force constants for the distance restraints were kept at 2 kcal mol^−1^ Å^−2^. The force constants for restraints involving non-exchangeable and exchangeable protons were then increased to 16 kcal mol^−1^ Å^−2^ and 8 kcal mol^−1^.Å^−2^ respectively in 20 ps before another equilibration at 1000 K for 50 ps. Next, the system was cooled down to 300 K in 42 ps, after which an equilibration was performed for 18 ps. The coordinates were saved every 0.5 ps during the last 10.0 ps and averaged. The average structure obtained was then subjected to minimization until the gradient of energy was l<0.1 kcal.mol^−1^. Dihedral (50 kcal mol^−1^ rad^−2^) and planarity (1 kcal mol^−1^ Å^−2^) restraints were maintained throughout the course of refinement. Ten lowest-energy structures were selected.

## RESULTS AND DISCUSSION

### Formation of two distinct QDH topologies in the *PIM1* gene

**Figure 1.**
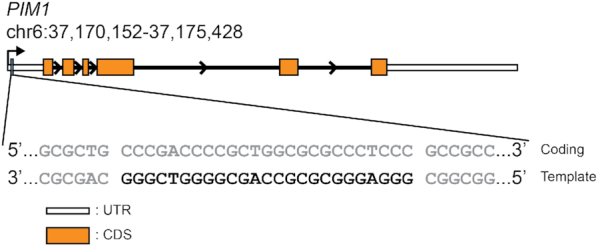
Genome architecture of the *PIM1* gene (RefSeqGene ID: NG_029601) and localization of the *PIM1* stem-loop-containing quadruplex sequence (SLQS). The *PIM1* gene locus on Chromosome 6 is shown, with the 5′- and 3′-untranslated regions (UTRs), coding sequence (CDS), and introns represented as white boxes, orange boxes, and black lines, respectively. The position of the *PIM1* SLQS (black typeface), which resides on the template strand, is demarcated and the genomic sequence in the vicinity (gray typeface) is shown.

**Figure 2.**
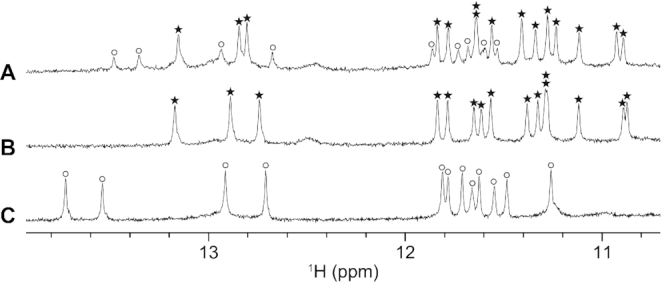
1D imino proton NMR spectra of (**A**) *PIM1-SLQS08*, (**B**) *PIM1-SLQS07* and (**C**) *PIM1-SLQS02*. Imino proton peaks corresponding to Form 1 and Form 2 QDH are labeled with stars and circles, respectively.

A stem-loop-containing quadruplex sequence (SLQS) was previously identified in the human *PIM1* gene near the transcription start site (Figure 1) and was shown to adopt multiple QDH topologies (37). Starting from the core sequence GGGAGGGCGCGCCAGCGGGGTCGGG (named *PIM1-SLQS01*), we performed a systematic sequence expansion from the 5′- and/or 3′-ends to dissect the potential structural species that can arise (Table 1 and Supplementary Table S1). 1D imino proton NMR spectra of these sequences showed either the presence of one QDH conformation or the other, a mixture of both forms, as well as additional conformations (Supplementary Figure S1). For instance, *PIM1-SLQS08* displayed one major (Form 1) and one minor (Form 2) conformations (Figure 2A), *PIM1-SLQS07* showed predominantly the presence of Form 1 (Figure 2B), while *PIM1-SLQS02* showed predominantly the presence of Form 2 (Figure 2C). Form 1 was characterized by twelve imino proton peaks at 10.8–11.9 ppm (Figure 2A, B), which are indicative of G-tetrad formation, and three imino proton peaks at 12.7–13.2 ppm, which are indicative of Watson–Crick base pair formation. These observations indicated that Form 1 corresponds to a QDH comprising a three-layered G-tetrad core and three Watson–Crick base pairs. On the other hand, Form 2 was characterized by eight G-tetrad imino proton peaks at 11.2–11.9 ppm and four Watson-Crick base pair imino proton peaks at 12.7–13.9 ppm (Figure 2A, C), indicating the formation of a QDH with a two-layered G-tetrad core and four Watson-Crick base pairs. The relative abundance of the two conformations in the series of *PIM1-SLQS01* derivatives suggested that Form 1 was favored by nucleotide extension from the 5′-end (*PIM1-SLQS07*) of the SLQS, while Form 2 was favored by nucleotide extension from the 3′-end (*PIM1-SLQS02*).

**Table 1.** Representative DNA sequences used in this study with estimated populations of the two QDH conformations at 25°C based on NMR spectra

Name	Sequence^a,b^	Form 1	Form 2	Others
*PIM1-SLQS08*	GC **GGG**A**GGG**CGCGCCAGC**GGGG**TC**GGG** C	>75%	∼20%	<5%
*PIM1-SLQS07*	GC **GGG**A**GGG**CGCGCCAGC**GGGG**TC**GGG**	>95%	–	<5%
*PIM1-SLQS02*	**GGG**A**GGG**CGCGCCAGC**GGGG**TC**GGG** C	–	>95%	<5%

^a^Tracts of contiguous guanines are shown in boldface. ^b^Complementary tracts are underlined.

### Gel electrophoretic shifts of Form 1 and Form 2 QDHs

Stoichiometry of the two QDH forms were probed by non-denaturing polyacrylamide gel electrophoresis (PAGE) (Supplementary Figure S2). A single major band was observed for both forms, consistent with their adoption of a single predominant structure. The bands for the two QDHs migrated at a comparable rate to that of a monomeric three-G-tetrad propeller-type all-parallel-stranded G4, but significantly faster than that of a dimeric interlocked G4 with a total of six G-tetrads, indicating that both QDHs are monomeric.

### CD study of Form 1 and Form 2 QDHs

**Figure 3.**
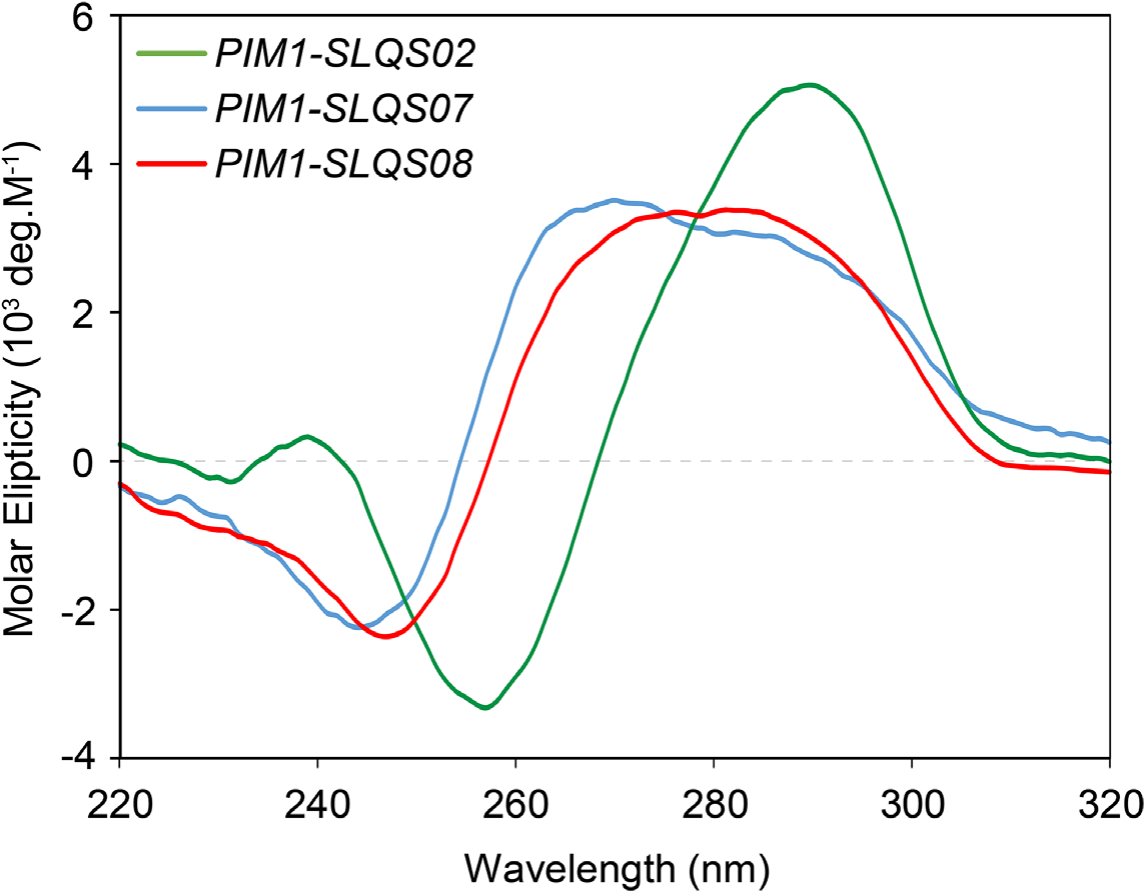
CD spectra of *PIM1-SLQS02* (Form 2; green), *PIM1-SLQS07* (Form 1; blue), and *PIM1-SLQS08* (Form 1/Form 2 mixture; red).

The CD spectrum of *PIM1-SLQS07*, which adopts predominantly Form 1, in K^+^ solution at 25°C showed a positive peak at ∼265 nm together with a positive shoulder at ∼290 nm, and a negative peak at ∼245 nm (Figure 3; blue curve). On the other hand, the CD spectrum of *PIM1-SLQS02*, which adopts predominantly Form 2, in K^+^ solution at 25°C showed a positive maximum at ∼290 nm and a negative minimum at ∼255 nm (Figure 3; green curve). These CD profiles are consistent with the G-tetrad core topologies of both Form 1 and Form 2 as determined by NMR (see below), which correspond to a (3+1) G-quadruplex and an anti-parallel G-quadruplex, respectively (46–48). CD melting of *PIM1-SLQS07* and *PIM1-SLQS02* were performed in 20 mM KCl and 20 mM potassium phosphate (pH 7), and showed a similar stability with the melting temperature of 65 and 67°C, respectively (Supplementary Figure S3). The CD spectrum of *PIM1-SLQS08*, which comprises a mixture of both Form 1 and Form 2, showed a broad positive band at ∼270–290 nm and a negative minimum at ∼245 nm (Figure 3, red curve). Using a linear fit as described previously (49), relative abundance of Form 1 and Form 2 in *PIM1-SLQS08* could be estimated based on the respective component spectrum. Using *PIM1-SLQS07* as the component spectrum for Form 1 and *PIM1-SLQS02* as the component spectrum for Form 2, we obtained a relative abundance of 81% and 19% for Form 1 and Form 2 in *PIM1-SLQS08*, respectively, consistent with the NMR observation (Table 1 and Figure 2A).

### NMR spectral assignments of Form 1 and Form 2 QDHs

We proceeded with the NMR structural characterization of Form 1 and Form 2 QDHs using *PIM1-SLQS07* and *PIM1-SLQS02*, respectively. The unambiguous assignments of selected guanine imino protons of both *PIM1-SLQS07* and *PIM1-SLQS02* (Supplementary Figure S4) were accomplished by site-specific low-enrichment ^15^N-labeling. Through-bond (COSY, TOCSY and ^13^C–^1^H–HSQC) and through-space (NOESY) correlation experiments facilitated the assignment of H8/H6-H1′ NOE sequential connectivity of the two constructs (Supplementary Figures S5 and S6) (50). For *PIM1-SLQS07*, the strong intensity of intra-residue H8-H1′ NOE cross-peaks for G3, G7, G20, G21 and G25 indicated their adoption of the *syn* glycosidic conformation (Supplementary Figure S5), while the remaining guanine residues adopt the *anti* glycosidic conformation. For *PIM1-SLQS02*, the strong intensity of intra-residue H8–H1′ NOE cross-peaks for G2, G6, G18 and G24 indicated their adoption of the *syn* glycosidic conformation (Supplementary Figure S6), while the remaining guanine residues adopt the *anti* glycosidic conformation. Relevant full-sized 1D and 2D NOESY spectra are shown in Supplementary Figures S7 and S8.

### Form 1 QDH is a (3+1) G-quadruplex

**Figure 4.**
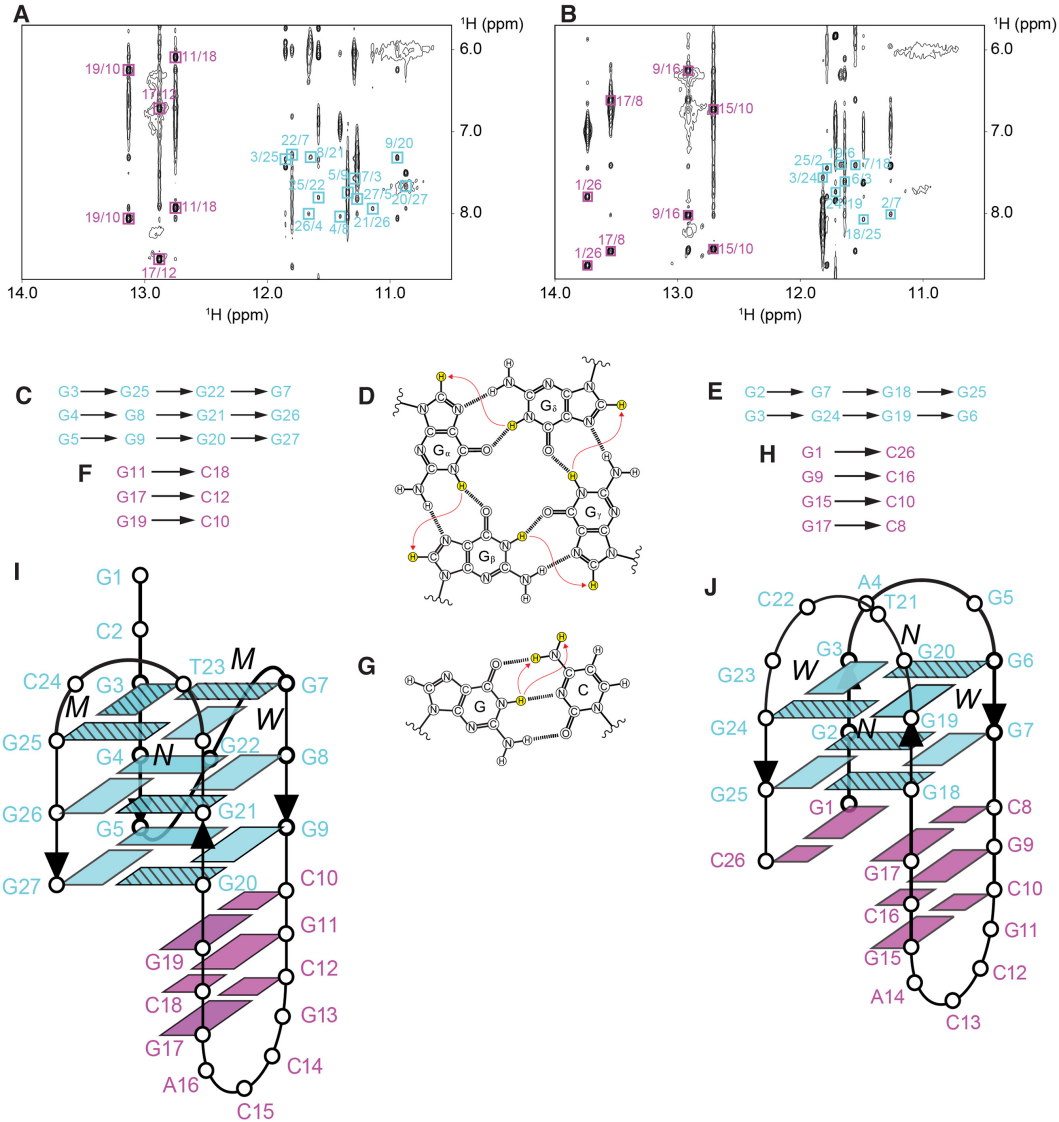
NOESY spectra (mixing time, 200 ms) showing the cross-peaks that establish the alignment of the G-tetrads (framed in cyan) and Watson–Crick base pairs (framed in magenta) in (**A**) Form 1 and (**B**) Form 2. (**C**) Cyclic guanine H1–H8 NOE connectivity patterns observed for G3•G25•G22•G7, G4•G8•G21•G26, and G5•G9•G20•G27 tetrads of Form 1, as outlined for the G_α_•G_β_•G_γ_•G_δ_ tetrad in (**D**). (**E**) Cyclic guanine H1–H8 NOE connectivity patterns observed for G2•G7•G18•G25 and G3•G24•G19•G6 tetrads of Form 2. (**F**) NOE connectivity patterns from guanine imino proton to cytosine amino protons observed for G11•C18, G17•C12 and G19•C10 base pairs of Form 1, as outlined for the Watson–Crick G•C base pair in (**G**). (**H**) NOE connectivity patterns from guanine imino proton to cytosine amino protons observed for G1•C26, G9•C16, G15•C10 and G17•C8 base pairs of Form 2, as outlined for the Watson–Crick G•C base pair in (**G**). Schematic diagrams of *PIM1* Form 1 and Form 2 QDHs are shown in (**I**) and (**J**), respectively, with the stripes on guanine bases indicating *syn* conformations. The wide (W), medium (M) and narrow (N) grooves are also indicated. For all panel, cyan and magenta indicate the G-quadruplex and duplex regions, respectively.

The (3+1) G-quadruplex topology of Form 1 was deduced based on cyclic imino-H8 NOE connectivity patterns around the individual G-tetrads (Figure 4A, D). The core consists of three G-tetrads, G3•G25•G22•G7, G4•G8•G21•G26 and G5•G9•G20•G27 (Figure 4C), with the relative hydrogen-bond directionality of the tetrads being anticlockwise-clockwise-clockwise, respectively (Figure 4I). The placement of the G4•G8•G21•G26 tetrad in the middle was supported by the slower rate of exchange of the imino protons from this G-tetrad with the solvent as compared to those of the other guanines of the G-tetrad core (Supplementary Figure S9). Signature Watson-Crick G•C base pair imino-amino NOE cross-peaks indicated the formation of three continuous base pairs G19•C10, G11•C18, and G17•C12 (Figure 4A, F, G).

**Figure 5.**
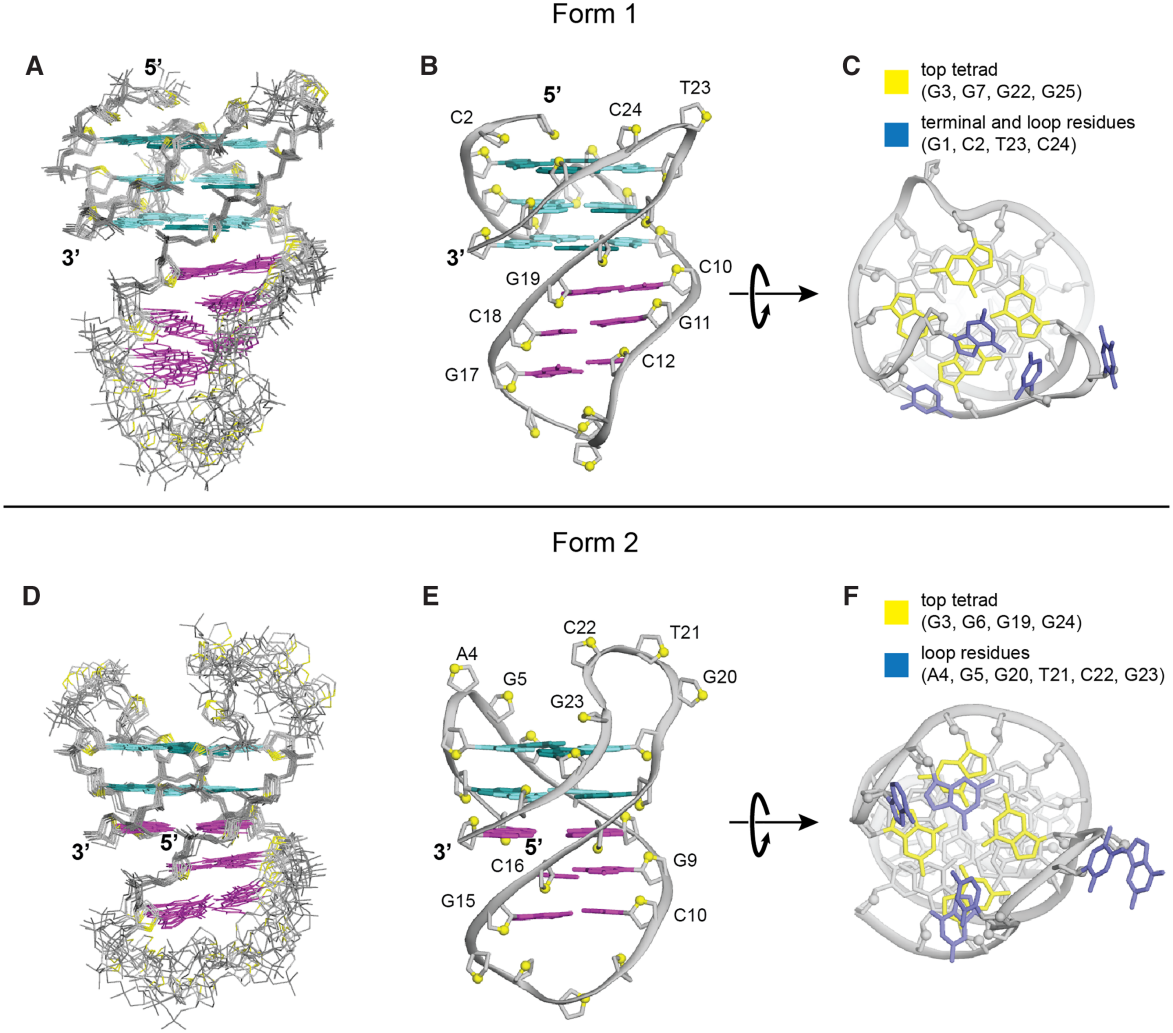
Solution structures of Form 1 (*PIM1-SLQS07*) and Form 2 (*PIM1-SLQS02*). (**A**, **D**) Superposition of the 10 lowest-energy structures, (**B**, **E**) best representative side view, and (**C**, **F**) top view of the two structures are presented. In panel A, B, D and E, cyan and magenta indicate G-quadruplex and duplex region, respectively. For panel C and F, only the top tetrad guanines (colored in yellow) and surrounding loop residues (colored in blue) are highlighted, while the rest are in gray color.

Structure calculation of Form 1 was performed using the following restraints: (i) distance restraints obtained from the three NOESY spectra (90%/10% H_2_O/D_2_O at 200 ms, 100% D_2_O at 100 and 300 ms), (ii) dihedral restraints for chi (χ) angles deduced from the intensity of intramolecular H8–H1′ cross-peaks, (iii) hydrogen-bond and (iv) planarity restraints formulated from the proposed base arrangements. Out of 100 calculated structures, the superposition of the 10 lowest-energy structures and the representative ribbon view are presented (Figure 5A–C). The structure calculation statistics are presented in Table 2. The solution structure confirmed the formation of a QDH as initially deduced. The duplex stem is capped by a four-nucleotide hairpin loop G13–C14–C15–A16. This hairpin stem is adjoined immediately across the wide groove of the G-tetrad core in a coaxial arrangement, with continuous stacking between the G19•C10 base pair and the bottom G-tetrad (Figure 4I). A6 adopts a single-nucleotide propeller loop configuration to connect G5 and G7 across a medium groove, while the two-nucleotide lateral loop T23–C24 folds back across a narrow groove to bridge G22 and G25. The 5′-terminal residue G1 was found to adopt *syn* conformation with well-defined convergence across all ten calculated structures, defined by multiple NOE cross-peaks observed between the sugar protons of G1 and the guanine imino protons in the top G-tetrad. The proximity between the G1 base and the opposing lateral loop residues (T23/C24) suggest possible interactions between them, although no direct evidence was observed, possibly due to the dynamic nature of the terminal residue.

**Table 2.** Statistics of the computed structures of Form 1 (*PIM1-SLQS07*) and Form 2 (*PIM1-SLQS02*)

	Form 1		Form 2	
A. NMR Restraints				
Distance restraints	D_2_O	H_2_O	D_2_O	H_2_O
Intra-residue	319	0	311	0
Inter-residue	147	64	151	58
Other restraints				
Hydrogen bond	66		56	
Dihedral angle	19		16	
Planarity	6		5	
B. Structure Statistics				
NOE violations				
Number (>0.2 Å)	0.900 ± 0.568		0.800 ± 0.789	
Deviations from the ideal geometry				
Bond lengths (Å)	0.003 ± 0.000		0.003 ± 0.000	
Bond angles (°)	0.698 ± 0.005		0.693 ± 0.009	
Impropers (°)	0.341 ± 0.004		0.340 ± 0.005	
Pairwise heavy atom RMSD value (Å)				
G-tetrad core	0.269 ± 0.021		0.309 ± 0.066	
All heavy atom	2.364 ± 0.345		2.732 ± 0.292	

### Form 2 QDH is a chair-type G-quadruplex with a G•C•G•C tetrad

For Form 2, characteristic cyclic imino-H8 NOE connectivity patterns around the individual G-tetrads (Figure 4B, D) pointed to the alternate alignment of the two G-tetrads, G2•G7•G18•G25 and G3•G24•G19•G6 (Figure 4E), into a chair-type (or antiparallel up-down-up-down) core topology (Figure 4J). Signature Watson–Crick G•C base pair imino-amino NOE cross-peaks indicated the formation of four base pairs G1•C26, G17•C8, G9•C16 and G15•C10 (Figure 4B, G, H), with the latter three form a continuous hairpin stem. The G17•C8 and G1•C26 base pairs are situated across two opposing wide grooves. They further aligned into a slipped G•C•G•C tetrad, supported by the observation of NOE cross-peaks between G1(H8) and C8(H41)/C8(H42)/C8(H5), and between G17(H8) and C26(H41)/C8(H42)/C8(H5) (Supplementary Figure S10). This is consistent with previous G•C•G•C tetrad-containing quadruplex structures formed in the presence of K^+^, which also showed a slipped alignment for the G•C•G•C tetrads (51). Formation of the G1•C26•G17•C8 tetrad was also consistent with the slower solvent exchange rate observed for the imino protons of G2, G7, G18, and G25 (Supplementary Figure S11). Similarly, the slower solvent exchange rate of G17 imino proton as compared to imino protons from the other G•C base pairs indicated the placement of the G17•C8 base pair within the G•C•G•C tetrads (Supplementary Figure S11).

Structure calculation of Form 2 was performed as described for Form 1 above. The superposition of the 10 lowest-energy structures and the representative ribbon view are presented (Figure 5D–F). The structure calculation statistics are presented in Table 2. The solution structure of Form 2 corroborated the proposed QDH fold. The duplex stem is capped by a four-nucleotide hairpin loop G11–C12–C13–A14 (Figure 4J). Similar to Form 1, the hairpin stem extends outward from the wide groove of the G-tetrad core in a coaxial arrangement, with continuous stacking between both the G17•C8 and G1•C26 base pairs and the bottom G-tetrad. The four bases consequently form a slipped G•C•G•C tetrad layer between the G-tetrad core and the duplex. The two other lateral loops, A4–G5 and G20–T21–C22–G23, traverse across opposite narrow grooves at the top end of the G-quadruplex (Figure 4J).

### Two coexisting QDH topologies and implications for drug targeting

We have shown that the *PIM1* SLQS near the transcription start site can adopt two distinct QDH topologies, which could coexist under the natural sequence context. Form 1 consists of a (3+1) G-tetrad core and a coaxially oriented duplex stem (Figure 4I), while Form 2 consists of a chair-type G-tetrad core stacked against a G•C•G•C tetrad, with a duplex stem further extending out from the latter in a coaxial arrangement (Figure 4J). The presence of the 3′ terminal C residue seems to favor Form 2 through its involvement in the formation of a G•C•G•C tetrad, while the presence of 5′-GC might disfavor this form by a possible clash with the duplex groove and/or favor Form 1 by possible interactions with the adjacent loop. Coexistence of two or more major quadruplex or QDH conformations within a single stretch of DNA have been observed across several G-rich promoter sequences, including that of *KIT* (52,53), *hTERT* (49,54), *KRAS* (55–58) and *EGFR* (59). Each of the coexisting isoforms could serve as a relevant therapeutic target, and might be targeted individually or in concert.

QDH complexes, having diverse structural features, offer multiple sites/avenues for targeting. The quadruplex element could be targeted by tetrad- and loop-binding ligands (60–73). For instance, specific to the two alternative structures of *PIM1* SLQS, each of the exposed tetrads has its unique properties (Figure 5C, F). The top tetrad of Form 1 is partially covered by the two 5′-terminal residues (G1 and C2) and two narrow groove lateral loop residues (T23 and C24), with all four localized on one site of the tetrad (Figure 5C). On the other hand, the top tetrad of Form 2 is covered by a total of six residues (A4–G5 and G20–G23) coming from both sites of the tetrad, bridging the two individual narrow grooves from opposite sites. There are two medium grooves and single wide and narrow grooves for Form 1, while there are pairs of wide and narrow grooves in alternating fashion in Form 2 (Figure 4I, J). These differences can potentially be exploited in designing selective ligands for the two forms.

**Figure 6.**
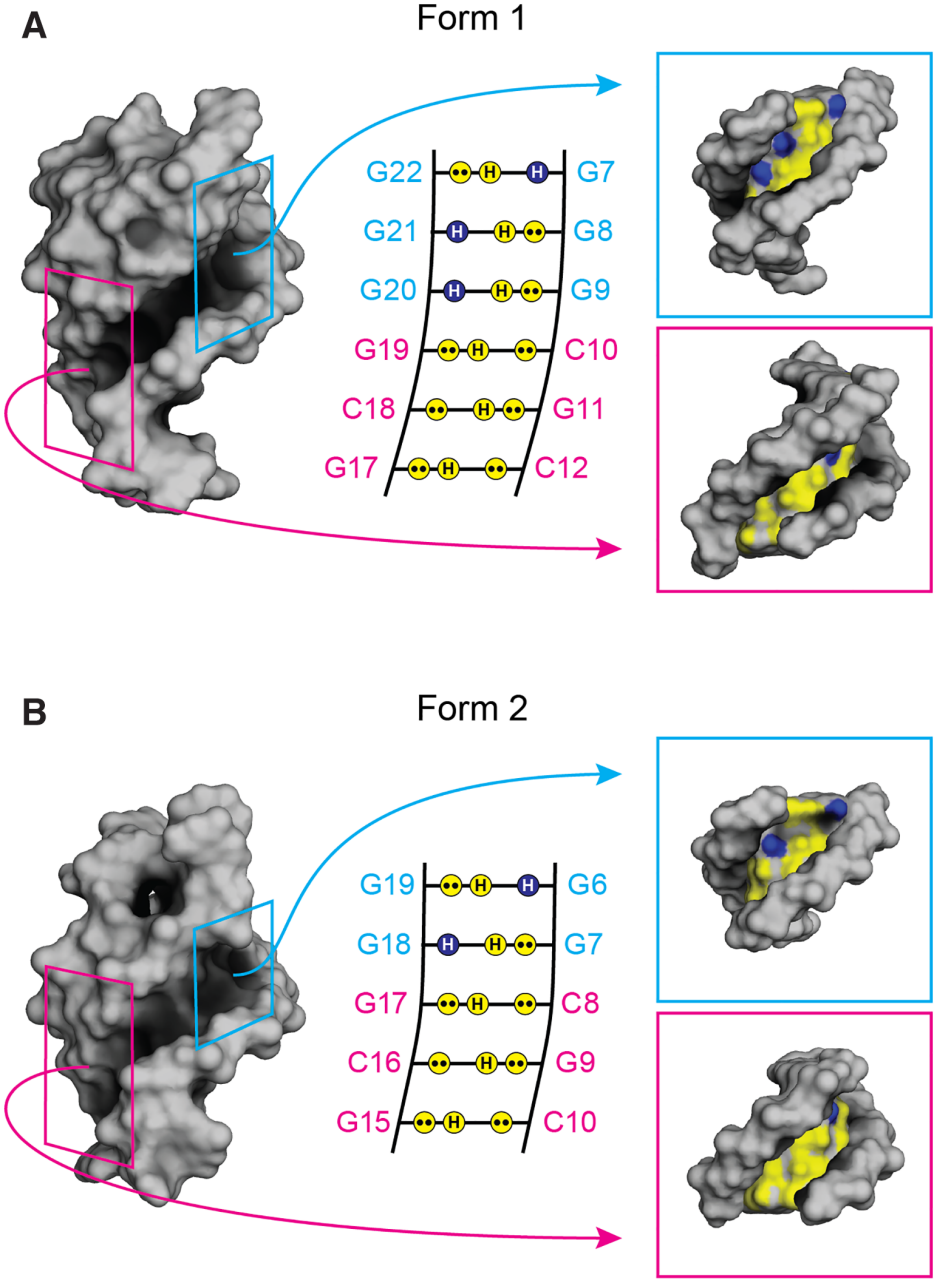
The groove progressions of the two *PIM1* QDH structures of (**A**) Form 1 and (**B**) Form 2. (Left) The surface representations. The quadruplex and duplex regions of the grooves are highlighted by the two boxes colored cyan and magenta, respectively. (Middle) The schematics representing the groove progressions of the two forms. Projections of lone pairs (••) from N3 of guanine or O6 of cytosine, and hydrogen (H) from N2 of guanine are polar and represented by yellow circles, while the hydrogen (H) from C8 of guanines are non-polar, indicated by blue circles. (Right) The individual surface representation of the quadruplex and duplex elements, with the same polar and non-polar color coding. The continuous grooves between the quadruplex and duplex elements of the QDHs can be selectively targeted by pyrrole–imidazole polyamide compounds.

The duplex element could be targeted by duplex- or stem-loop-binding agents (74), thus providing the opportunity for sequence-specific recognition (75–79). Interestingly, the tetrad- and duplex-binding ligands could also be combined to achieve synergistic binding of QDH structures for enhanced selectivity (80–82). Of particular interest is the quadruplex–duplex junction, which would provide a unique interface for ligand targeting (34,83,84). Conceptually, the two alternative QDH structures of *PIM1* SLQS would be excellent targets for the pyrrole–imidazole polyamide (PIP) class of duplex minor groove-binding agents (76,78,79); the duplex stem and G-tetrad core are stacked against each other, thus presenting a continuous progression of the groove for accommodation of a ligand (Figure 6). PIPs have been successfully designed to selectively target Watson-Crick A•T, T•A, G•C and C•G base pair steps on the minor groove of a duplex stem. For the G-tetrad groove recognition, this could potentially be addressed by a combination of building blocks that selectively recognize G→G and G←G steps. In the context of Form 1, from top (quadruplex) to bottom (duplex), the base pairings are respectively, G22→G7, G21←G8, G20←G9, G19•C10, C18•G11 and G17•C12 (Figure 6a). Its progression involves three layers of exposed non-polar protons (cyan box, Figure 6A) followed by three layers of standard G•C pairs (magenta box, Figure 6a). For Form 2, the base pairings are respectively, G19→G6, G18←G7, G17•C8, C16•G9 and G15•C10 (Figure S6b). It starts from two layers of exposed non-polar protons (cyan box, Figure 6B) followed by the same three layers of standard G•C pairs (magenta box, Figure 6B). The subtle differences could potentially be exploited in specific ligand design. Such a targeting strategy would thus provide a straightforward route towards the specific recognition of a quadruplex groove. The two *PIM1* QDH structures, localized on the template strand near the transcription start site, hence represent attractive targets for the downregulation of *PIM1* expression through inhibition of its transcriptional activity.

## CONCLUSION

The SLQS found in the *PIM1* gene was shown to adopt two distinct QDH conformations. The solution structures of the two coexisting QDH structures were solved by NMR spectroscopy: Form 1 was found to adopt a (3+1) G-tetrad core with a propeller loop, a coaxially-stacked hairpin stem-loop, and a lateral loop; Form 2 was found to adopt a chair-type G-tetrad core and an adjoining G•C•G•C tetrad, with two lateral loops and a coaxially-stacked hairpin stem-loop. These QDH structures represent attractive targets for ligand design towards the downregulation of the *PIM1* gene for anticancer treatment.

## DATA AVAILABILITY

The coordinates of the structures of Form 1 and Form 2 QDHs in the *PIM1* gene have been deposited in the Protein Data Bank (PDB codes: 7CV3 and 7CV4)

## Supplementary Material

gkaa752_Supplemental_File
