# Supplementary material for: Coexistence of two quadruplex–duplex hybrids in the PIM1 gene
Source: Nucleic Acids Res. 2020 Sep 25;48(19):11162–71. doi: 10.1093/nar/gkaa752 (PMC7641742; doi:10.1093/nar/gkaa752)
Supplement: gkaa752_Supplemental_File [file gkaa752_supplemental_file.docx]

**Coexistence of Two Quadruplex-Duplex Hybrids in the *PIM1* Gene**

Derrick J. Y. Tan^1,†^, Fernaldo Richtia Winnerdy^1,†^, Kah Wai Lim,^1^ and Anh Tuân Phan*^,1,2^

^1^School of Physical and Mathematical Sciences, Nanyang Technological University, Singapore 637371, Singapore

^2^NTU Institute of Structural Biology, Nanyang Technological University, Singapore 636921, Singapore

**Supplementary Materials**

**Table S1.** DNA sequences used in this study.

| Name | Sequence^a,b^ |
| --- | --- |
| *PIM1-SLQS01* | **GGG**A**GGG**CGCGCCAGC**GGGG**TC**GGG** |
| *PIM1-SLQS02* | **GGG**A**GGG**CGCGCCAGC**GGGG**TC**GGG** C |
| *PIM1-SLQS03* | **GGG**A**GGG**CGCGCCAGC**GGGG**TC**GGG** CA |
| *PIM1-SLQS04* | C **GGG**A**GGG**CGCGCCAGC**GGGG**TC**GGG** |
| *PIM1-SLQS05* | C **GGG**A**GGG**CGCGCCAGC**GGGG**TC**GGG** C |
| *PIM1-SLQS06* | C **GGG**A**GGG**CGCGCCAGC**GGGG**TC**GGG** CA |
| *PIM1-SLQS07* | GC **GGG**A**GGG**CGCGCCAGC**GGGG**TC**GGG** |
| *PIM1-SLQS08* | GC **GGG**A**GGG**CGCGCCAGC**GGGG**TC**GGG** C |
| *PIM1-SLQS09* | GC **GGG**A**GGG**CGCGCCAGC**GGGG**TC**GGG** CA |

^a^Tracts of contiguous guanines are shown in boldface. ^b^Complementary tracts are underlined.


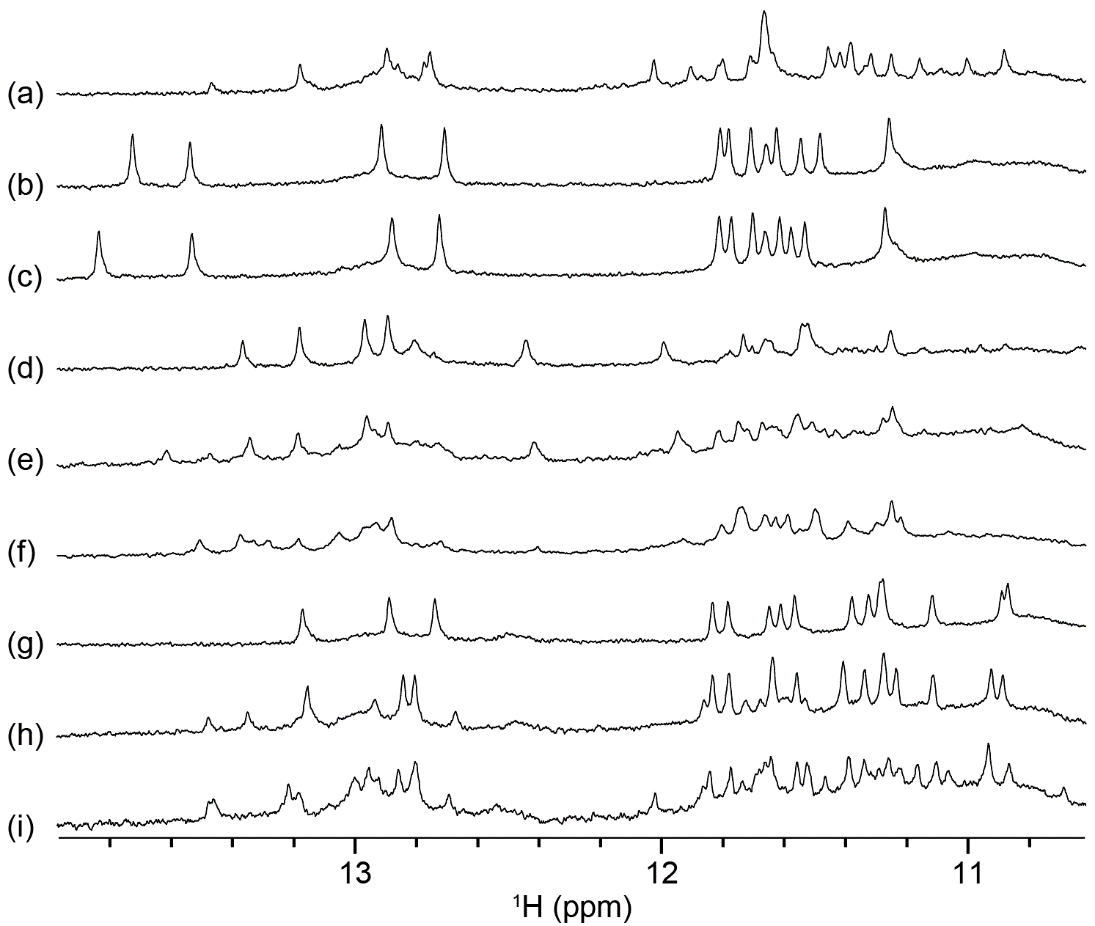


**Figure S1**. 1D imino proton NMR spectra of (a) *PIM1-SLQS01*, (b) *PIM1-SLQS02*, (c) *PIM1-SLQS03*, (d) *PIM1-SLQS04*, (e) *PIM1-SLQS05*, (f) *PIM1-SLQS06*, (g) *PIM1-SLQS07*, (h) *PIM1-SLQS08*, and (i) *PIM1-SLQS09*.


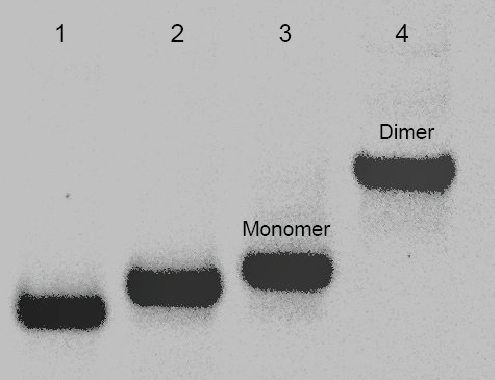


**Figure S2**. Non-denaturing PAGE of *PIM1-SLQS02* (Form 2; Lane 1) and *PIM1-SLQS07* (Form 1; Lane 2), as compared to a monomeric (three-layered) G4 (d[TT(GGGT)_4_] (1); Lane 3) and a dimeric (six-layered) G4 (d[GGGGTGGGAGGAGGGT] (2); Lane 4) markers.


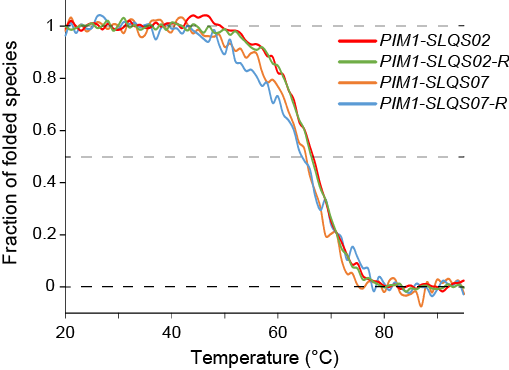


**Figure S3**. Melting of *PIM1-SLQS02* and *PIM1-SLQS07* monitored by CD signal at 295 nm. For each sequence, both heating and cooling curves are shown.


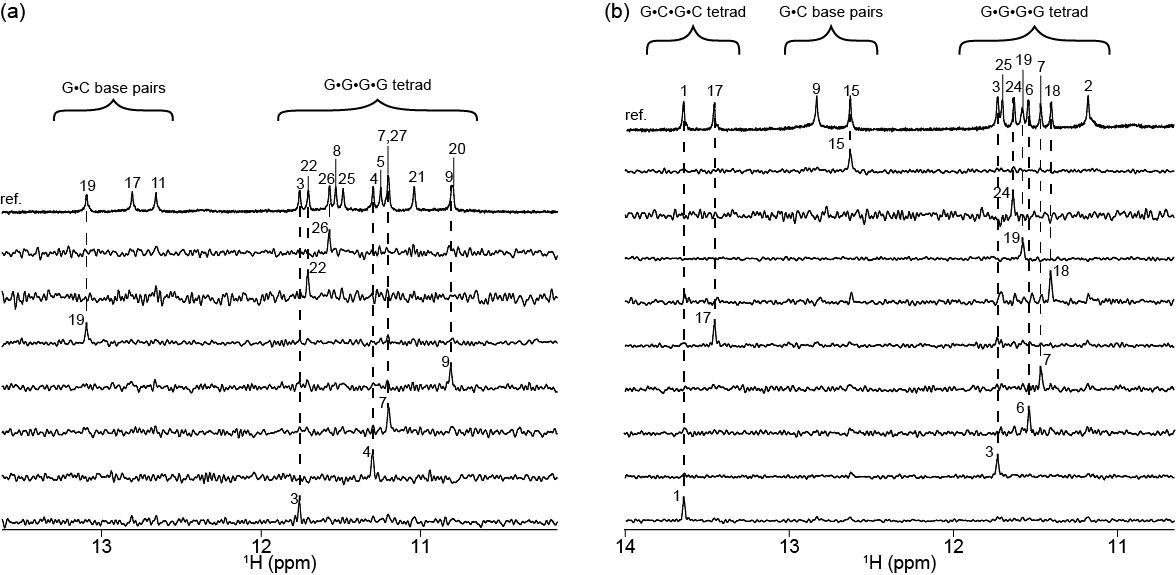


**Figure S4**. Assignment of G-tetrad and Watson-Crick imino protons of (a) Form 1 and (b) Form 2 *PIM1* quadruplex-duplex hybrid through ^15^N-filtered spectra of selected samples, 2% ^15^N-labeled at the indicated positions.


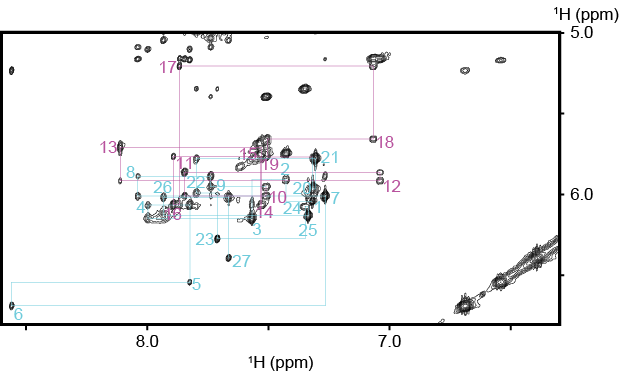


**Figure S5.** NOESY spectrum (mixing time, 300 ms) showing H8/H6-H1ʹ sequential connectivity of *PIM1-SLQS07* (Form 1). Intraresidue H8/H6-H1ʹ cross-peaks are labeled with residue numbers.


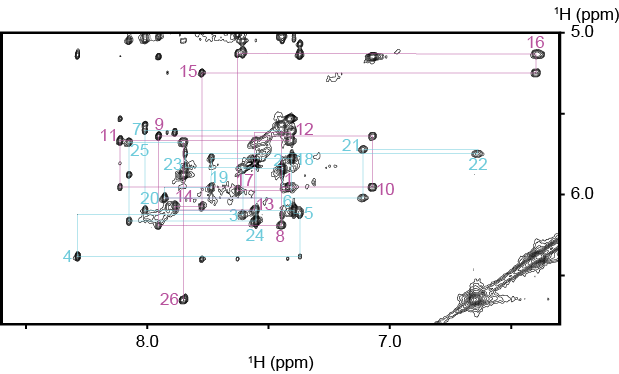


**Figure S6.** NOESY spectrum (mixing time, 300 ms) showing H8/H6-H1ʹ sequential connectivity of *PIM1-SLQS02* (Form 2). Intraresidue H8/H6-H1ʹ cross-peaks are labeled with residue numbers.

**
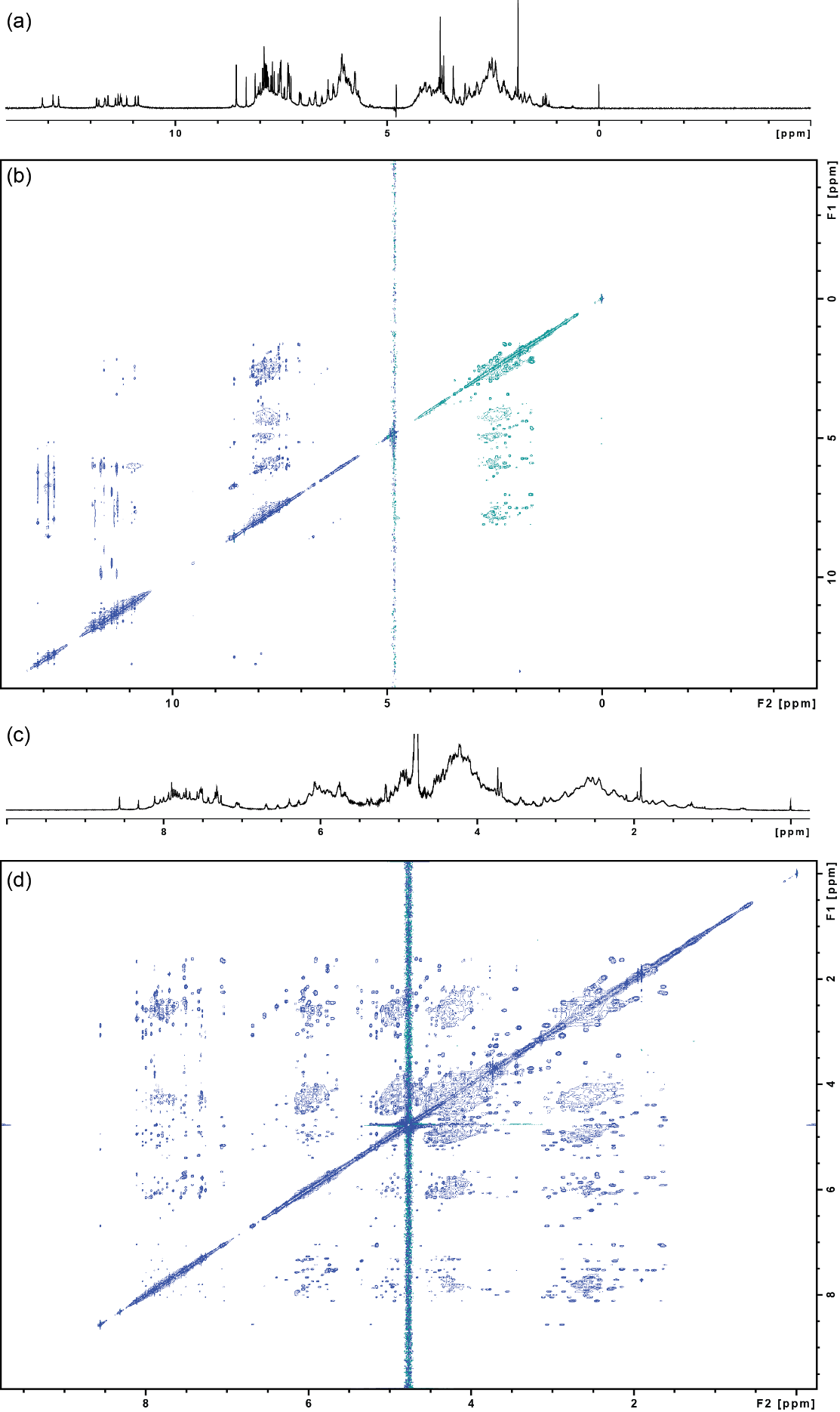
**

**Figure S7.** Full-size spectra of *PIM1-SLQS07* (Form 1). (a) 1D spectrum in 90%/10% H_2_O/D_2_O with Watergate water suppression, (b) 2D spectrum in 90%/10% H_2_O/D_2_O with jump-and-return echo water suppression scheme, (c) 1D spectrum in 100% D_2_O and (d) 2D spectrum in 100% D_2_O.


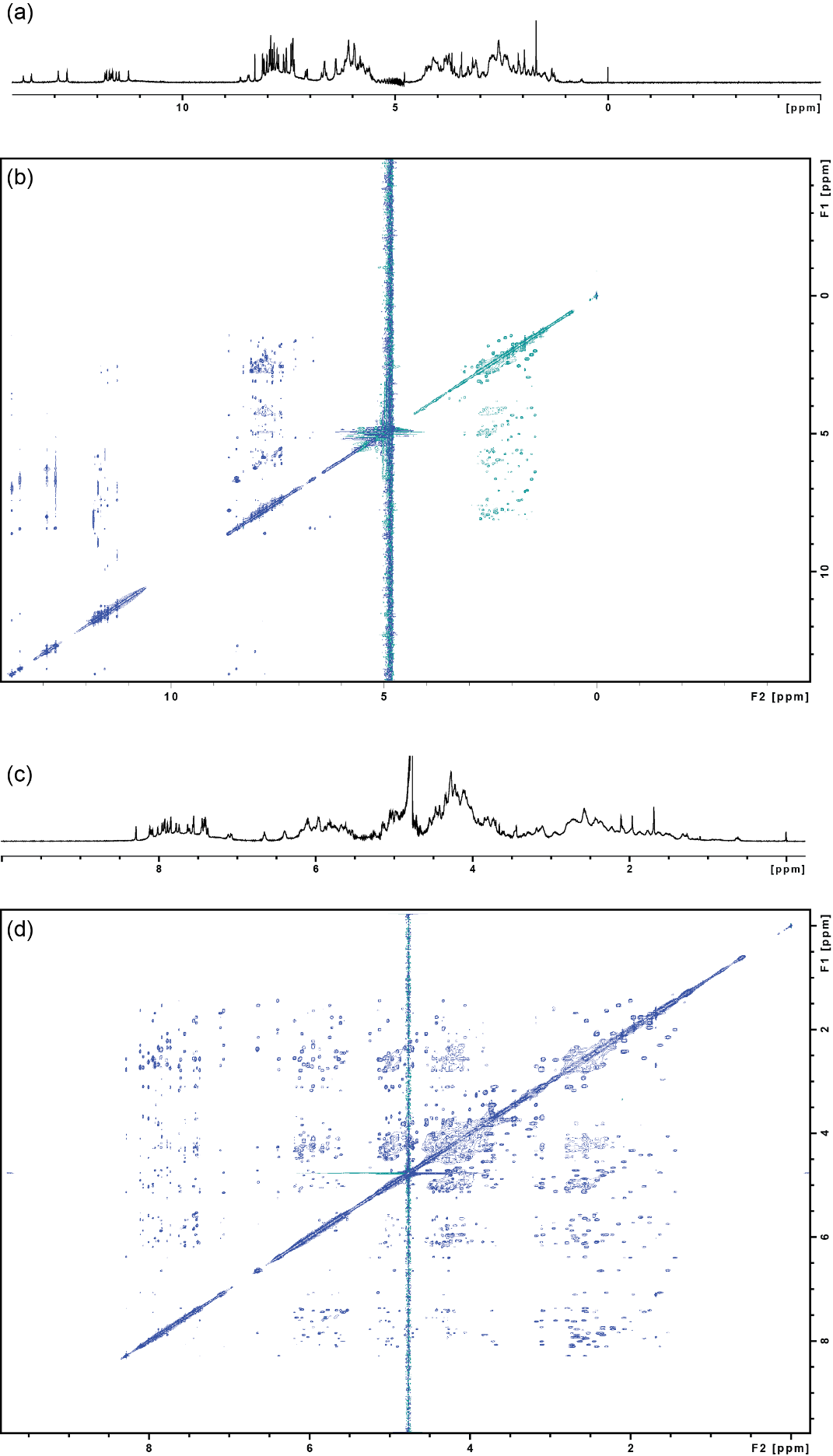


**Figure S8.** Full-size spectra of *PIM1-SLQS02* (Form 2). (a) 1D spectrum in 90%/10% H_2_O/D_2_O with Watergate water suppression, (b) 2D spectrum in 90%/10% H_2_O/D_2_O with jump-and-return echo water suppression scheme, (c) 1D spectrum in 100% D_2_O and (d) 2D spectrum in 100% D_2_O.


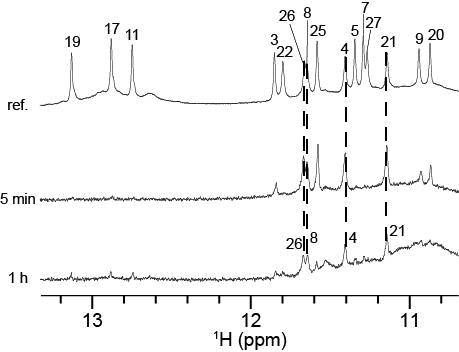


**Figure S9.** 1D imino proton NMR spectrum of *PIM1-SLQS07* (Form 1) after 5 mins and 1 h in D_2_O at 25 °C.


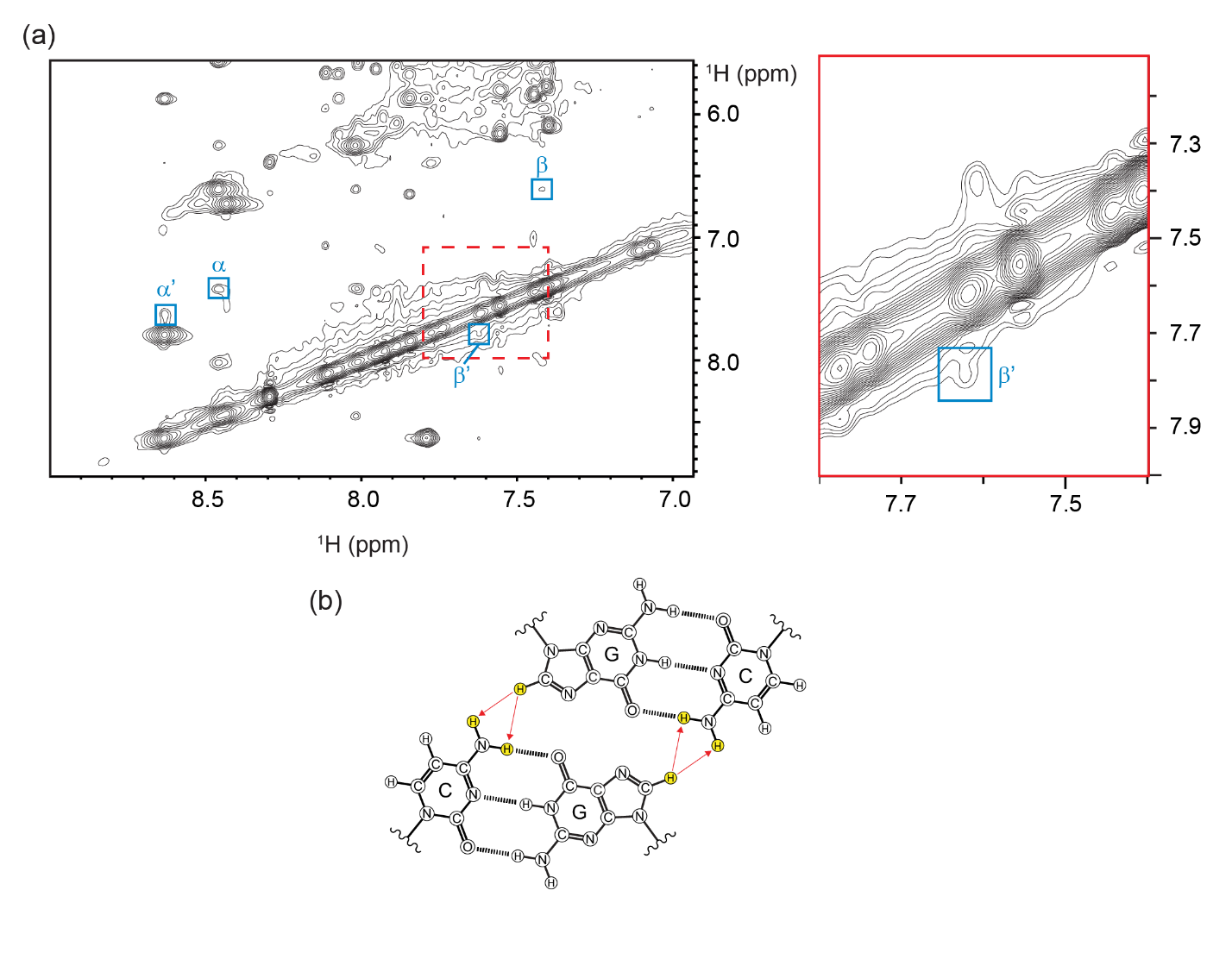


**Figure S10.** (a) NOESY spectrum (mixing time, 200 ms) showing the cross-peaks corresponding to the slipped G•C•G•C tetrad arrangement. The inset (marked by red box) shows the diagonal region with higher threshold to show the existence of the cross-peak βʹ more clearly. The NOE cross-peaks of G1(H8)-C8(H41), G17(H8)-C26(H41), G1(H8)-C8(H42) and G17(H8)-C26(H42) are indicated by α, αʹ, β and βʹ respectively. (b) Schematic of the slipped G•C•G•C tetrad.


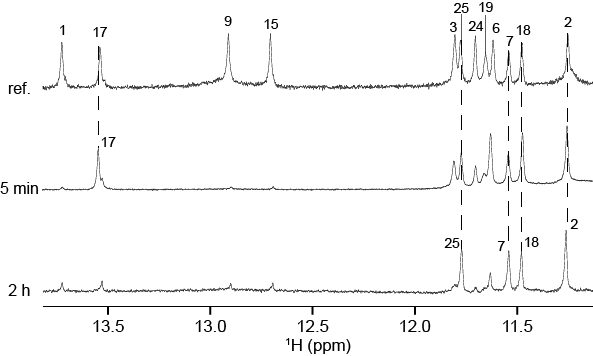


**Figure S11.** 1D imino proton NMR spectrum of *PIM1-SLQS02* (Form 2) after 5 mins and 2 h in D_2_O at 25 °C.

**REFERENCES**

1. Do, N.Q. and Phan, A.T. (2012) Monomer-dimer equilibrium for the 5 '-5 ' stacking of propeller-type parallel-stranded G-quadruplexes: NMR structural study. *Chem. Eur. J.*, **18**, 14752-14759.

2. Phan, A.T., Kuryavyi, V., Ma, J.B., Faure, A., Andreola, M.L. and Patel, D.J. (2005) An interlocked dimeric parallel-stranded DNA quadruplex: a potent inhibitor of HIV-1 integrase. *Proc Natl Acad Sci U S A*, **102**, 634-639.
